# Supplementary material for: The Evolution and Characterization of the RNA Interference Pathways in Lophotrochozoa
Source: Genome Biol Evol. 2024 May 7;16(5):evae098. doi: 10.1093/gbe/evae098 (PMC11114477; doi:10.1093/gbe/evae098)
Supplement: evae098_Supplementary_Data [file evae098_supplementary_data.zip › Supplementary_materials.zip!/selected_miRNAs/NoGe_selected_miRNAs/mirdeep_pdf_files/NMRB01002279.1_Notospermus_geniculatus_strain_Ushimado_scaffold2279,_whole_genome_shotgun_sequence_37112.pdf]

Provisional ID	:	NMRB01002279.1_Notospermus_geniculatus_strain_Ushimado_scaffold2279,_whole_genome_shotgun_sequence_37112
Score total	:	47237.3
Score for star read(s)	:	3.9
Score for read counts	:	47229.3
Score for mfe	:	1.1
Score for randfold	:	0
Score for cons. seed	:	3
Total read count	:	92650
Mature read count	:	92152
Loop read count	:	0
Star read count	:	498

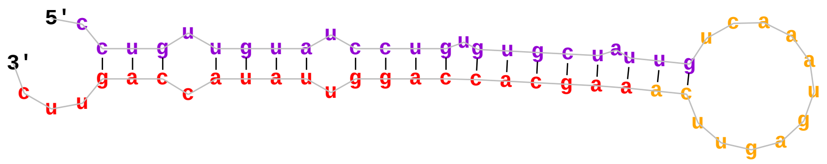

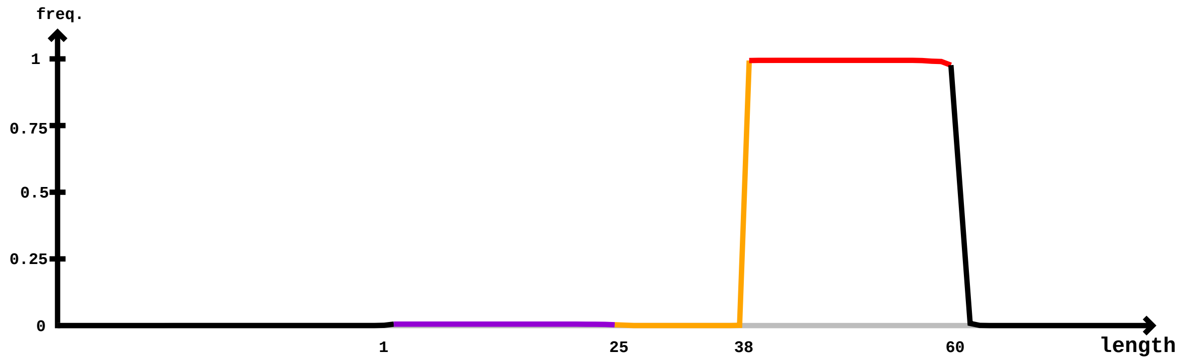


```
                                                 Star                               Mature
5'- uagaaguuguuuugucggauugcugcccagugcccuguuguauccugugugcuauugucaaaugaguucaaagcaccagguuauaccaguucauuuggcaugcaaauaaaua   -3'    obs
    uagaaguuguuuugucggauugcugcccagugcccuguuguauccugugugcuauugucaaaugaguucaaagcaccagguuauaccaguucauuuggcaugcaaauaaaua          exp
    ...................((((((((.((((..(((.((((.((((.(((((.(((...........)))))))))))).)))).)))..)))).)))).))))....... reads   mm   sample
    .................gauuUcugcccagugccc............................................................................. 1       1    Mm2
    .................gauuUcugcccagugcccu............................................................................ 2       1    Mm2
    ................................cccuguuguauccugugugcu........................................................... 1       0    Mm2
    ................................cccuguuguauccugugugcuU.......................................................... 1       1    Mm2
    ................................cccuguuguauccugugugcuau......................................................... 1       0    Mm2
    ................................cccuguuguauccugugugcuauug....................................................... 1       0    Mm2
    .................................ccuguuguauccugugugcu........................................................... 1       0    Mm2
    .................................ccuguuguauccugugugcua.......................................................... 1       0    Mm2
    .................................ccuguuguauccugugugcuau......................................................... 2       0    Mm2
    .................................ccuguuguauccugugugcuauA........................................................ 1       1    Mm2
    .................................ccuguuguauccugugugcuauu........................................................ 25      0    Mm2
    .................................ccuUuuguauccugugugcuauug....................................................... 2       1    Mm2
    .................................ccuguuguauccugugugcuauug....................................................... 57      0    Mm2
    .................................ccuguuguauccugugugcuauugA...................................................... 1       1    Mm2
    .........................................................ucaaaugaguucaaagcaccagguu.............................. 2       0    Mm2
    .....................................................................Caagcaccagguuauaccaguuc.................... 23      1    Mm2
    .....................................................................aaagcaccagguuauaccaguuc.................... 13      0    Mm2
    .....................................................................Caagcaccagguuauaccaguuca................... 1       1    Mm2
    ......................................................................aagcaccagguuauacca........................ 11      0    Mm2
    ......................................................................aagcaccagguuauaccaU....................... 1       1    Mm2
    ......................................................................aagcaccagguuauaccUg....................... 1       1    Mm2
    ......................................................................aagcaccagguuauaccag....................... 10      0    Mm2
    ......................................................................aagcaccagguuauaccUgu...................... 1       1    Mm2
    ......................................................................aagcaccagguuauaccagu...................... 36      0    Mm2
    ......................................................................aagcaccagguuauaccUguu..................... 1       1    Mm2
    ......................................................................aagcaccagAuuauaccaguu..................... 1       1    Mm2
    ......................................................................aagcaccagguuauaccaguu..................... 346     0    Mm2
    ......................................................................aagcaccagguuauacUaguu..................... 1       1    Mm2
    ......................................................................aagcaccaggGuauaccaguu..................... 1       1    Mm2
    ......................................................................aagcaccagguuauacAaguu..................... 4       1    Mm2
    ......................................................................aagcaccagguuauaccGguu..................... 1       1    Mm2
    ......................................................................aagcaccagguuauaccCguu..................... 2       1    Mm2
    ......................................................................aagcaccaggAuauaccaguu..................... 1       1    Mm2
```

```
                                Star                                 Mature
uagaaguuguuuugucggauugcugcccagugcccuguuguauccugugugcuauugucaaaugaguucaaagcaccagguuauaccaguucauuuggcaugcaaauaaaua
......................................................................aagcaccagguuauacAaguuc....................   174     1     Mm2
......................................................................aaAcaccagguuauaccaguuc....................   9       1     Mm2
......................................................................Gagcaccagguuauaccaguuc....................   8       1     Mm2
......................................................................aagcaccagguuauGccaguuc....................   4       1     Mm2
......................................................................aagcaccCgguuauaccaguuc....................   1       1     Mm2
......................................................................aagcaccagguuCuaccaguuc....................   2       1     Mm2
......................................................................aagcaccagguuauaccCguuc....................   134     1     Mm2
......................................................................aagcaccagguuauaccaCuuc....................   1       1     Mm2
......................................................................Cagcaccagguuauaccaguuc....................   3       1     Mm2
......................................................................aaUcaccagguuauaccaguuc....................   9       1     Mm2
......................................................................aagcaccagguuauaccaguuA....................   8       1     Mm2
......................................................................aagcaccaUguuauaccaguuc....................   4       1     Mm2
......................................................................aagGaccagguuauaccaguuc....................   2       1     Mm2
......................................................................aagcaccagguAauaccaguuc....................   3       1     Mm2
......................................................................Nagcaccagguuauaccaguuc....................   21      1     Mm2
......................................................................aCgcaccagguuauaccaguuc....................   2       1     Mm2
......................................................................aagcaccagguuauacGaguuc....................   53      1     Mm2
......................................................................aagcCccagguuauaccaguuc....................   3       1     Mm2
......................................................................aagcaccagUuuauaccaguuc....................   4       1     Mm2
......................................................................aagcaccagguGauaccaguuc....................   3       1     Mm2
......................................................................aagcaccagguCauaccaguuc....................   1       1     Mm2
......................................................................aagcaccagguuauaccUguuc....................   112     1     Mm2
......................................................................aagcaNcagguuauaccaguuc....................   5       1     Mm2
......................................................................aagcaccagguuauaccaAuuc....................   3       1     Mm2
......................................................................aagcaccagguuaAaccaguuc....................   8       1     Mm2
......................................................................aagcaccUgguuauaccaguuc....................   5       1     Mm2
......................................................................aagcaccaCguuauaccaguuc....................   1       1     Mm2
......................................................................aagcaccagguuauaccaUuuc....................   5       1     Mm2
......................................................................aagcaccagguuUuaccaguuc....................   6       1     Mm2
......................................................................aUgcaccagguuauaccaguuc....................   9       1     Mm2
......................................................................aagcaccaggCuauaccaguuc....................   39      1     Mm2
......................................................................aGgcaccagguuauaccaguuc....................   11      1     Mm2
......................................................................aagcaccagguuaCaccaguuc....................   8       1     Mm2
......................................................................aagcaccagguuauacUaguuc....................   101     1     Mm2
......................................................................aagcaccagguuauaccagCuc....................   3       1     Mm2
......................................................................aagcUccagguuauaccaguuc....................   1       1     Mm2
......................................................................aagcacNagguuauaccaguuc....................   2       1     Mm2
......................................................................aagcaccGgguuauaccaguuc....................   23      1     Mm2
......................................................................aaCcaccagguuauaccaguuc....................   4       1     Mm2
......................................................................aagcaccagAuuauaccaguuc....................   11      1     Mm2
......................................................................aagcaUcagguuauaccaguuc....................   9       1     Mm2
......................................................................aagcaccagguuauaccagAuc....................   5       1     Mm2
......................................................................aagcaccagguuauUccaguuc....................   7       1     Mm2
......................................................................aagcaccagguuGuaccaguuc....................   4       1     Mm2
......................................................................aagcacUagguuauaccaguuc....................   13      1     Mm2
......................................................................aagcaccagguuauaccaguuc....................   39840   0     Mm2
......................................................................Uagcaccagguuauaccaguuc....................   21      1     Mm2
......................................................................aagcGccagguuauaccaguuc....................   4       1     Mm2
......................................................................aagcacAagguuauaccaguuc....................   1       1     Mm2
......................................................................aagcaccagguuauaccaguuU....................   22      1     Mm2
......................................................................aagcaccagguuauaccaguAc....................   1       1     Mm2
......................................................................aagcaccagCuuauaccaguuc....................   1       1     Mm2
......................................................................aagcaGcagguuauaccaguuc....................   1       1     Mm2
......................................................................aagAaccagguuauaccaguuc....................   10      1     Mm2
......................................................................aagUaccagguuauaccaguuc....................   6       1     Mm2
......................................................................aagcaccaggAuauaccaguuc....................   77      1     Mm2
......................................................................aagcaccagguuaGaccaguuc....................   1       1     Mm2
......................................................................aagcaccagguuauaUcaguuc....................   9       1     Mm2
......................................................................aagcaccagguuauaccGguuc....................   111     1     Mm2
......................................................................aagcaccaggGuauaccaguuc....................   50      1     Mm2
......................................................................aagcaccagguuauaccaguCc....................   5       1     Mm2
......................................................................aagcaccagguuauaccaguuUa...................   1       1     Mm2
......................................................................aagcaccagguuauacAaguuca...................   13      1     Mm2
......................................................................aagcaccaggGuauaccaguuca...................   2       1     Mm2
......................................................................aagcaccagguuauaccaguuca...................   2451    0     Mm2
......................................................................aagcaccaggCuauaccaguuca...................   5       1     Mm2
......................................................................aagcacUagguuauaccaguuca...................   1       1     Mm2
......................................................................aagcaccagguuauacGaguuca...................   4       1     Mm2
......................................................................aagcaccagguuauaccCguuca...................   7       1     Mm2
......................................................................aagcaccagguuauacUaguuca...................   2       1     Mm2
```

```
                                Star                                Mature
uagaaguuguuuugucggauugcugcccagugcccuguuguauccugugugcuauugucaaaugaguucaaagcaccagguuauaccaguucauuuggcaugcaaauaaaua
......................................................................Nagcaccagguuauaccaguuca...................   1     1   Mm2
......................................................................aagcaccagguuauaccGguuca...................   9     1   Mm2
......................................................................aagcaccagguuauaccaguucU...................   1552  1   Mm2
......................................................................aaAcaccagguuauaccaguuca...................   1     1   Mm2
......................................................................aagcaccGgguuauaccaguuca...................   1     1   Mm2
......................................................................aagcaNcagguuauaccaguuca...................   2     1   Mm2
......................................................................aagcaccagguuauaUcaguuca...................   1     1   Mm2
......................................................................aagcaccagguuauaccaguucC...................   67    1   Mm2
......................................................................aagcaccagguuauaccUguuca...................   7     1   Mm2
......................................................................aagcaccaggAuauaccaguuca...................   5     1   Mm2
......................................................................aagcacNagguuauaccaguuca...................   1     1   Mm2
......................................................................aagcaccagguuauUccaguucau..................   1     1   Mm2
......................................................................aagcaccagguuauaccaguucaG..................   10    1   Mm2
......................................................................aagcaccagguuauaccGguucau..................   1     1   Mm2
......................................................................aagcaccagguuauaccaguucaC..................   10    1   Mm2
......................................................................aagcaccagguuauaccUguucau..................   1     1   Mm2
......................................................................aagcaAcagguuauaccaguucau..................   1     1   Mm2
......................................................................aagcaccagguuauaccaguucaA..................   112   1   Mm2
......................................................................aagcaccagguuauaccaguucUu..................   38    1   Mm2
......................................................................aagcaccagguuauaccaguucau..................   76    0   Mm2
......................................................................aGgcaccagguuauaccaguucau..................   1     1   Mm2
......................................................................aagcaccagguuauaccaguucaCu.................   2     1   Mm2
......................................................................aagcaccagguuauaccaguucaAu.................   7     1   Mm2
......................................................................aagcaccagguuauaccaguucauu.................   11    0   Mm2
......................................................................aagcaccagguuauaccaguucUuu.................   3     1   Mm2
......................................................................aagcaccagguuauaccaguucauuu................   1     0   Mm2
.......................................................................agcaccagguuauaccaguu.....................   1     0   Mm2
.......................................................................agcacUagguuauaccaguuc....................   1     1   Mm2
.......................................................................aAcaccagguuauaccaguuc....................   1     1   Mm2
.......................................................................agcaccagguuauaccaguuc....................   26    0   Mm2
.......................................................................agcaccagguuauaccaguucU...................   2     1   Mm2
.......................................................................agcaccagguuauaAcaguuca...................   1     1   Mm2
........................................................................gcaccagguuauaccaguu.....................   1     0   Mm2
.........................................................................caccagguuauaccaguuc....................   1     0   Mm2

.................................ccuguuguauccugugugcuau.........................................................   17    0   Mm1
.................................ccuguuguauccuguguUcuau.........................................................   1     1   Mm1
.................................ccuguuguauccugugugcuauu........................................................   7     0   Mm1
.................................ccuguuguauccugugugcuauug.......................................................   44    0   Mm1
.................................ccuguuguauccuguguUcuauug.......................................................   2     1   Mm1
.................................ccuguuguauGcugugugcuauug.......................................................   1     1   Mm1
...................................uguuguauccugugugcuauug.......................................................   4     0   Mm1
......................................................................aagcaccagguuauacca........................   19    0   Mm1
......................................................................aagcaccagguuauaccCg.......................   1     1   Mm1
......................................................................aagcaccagguuauaccag.......................   5     0   Mm1
......................................................................aagcaccagguuauaccagu......................   6     0   Mm1
......................................................................aagcaccaggGuauaccaguu.....................   1     1   Mm1
......................................................................aagcaccagguuauacAaguu.....................   2     1   Mm1
......................................................................aagcaccagguuauaccaguu.....................   255   0   Mm1
......................................................................aagcacUagguuauaccaguu.....................   1     1   Mm1
......................................................................aagcaccagguuauaccGguu.....................   1     1   Mm1
......................................................................aagcaccagguuauacGaguu.....................   1     1   Mm1
......................................................................aagcaccagguuauaccCguu.....................   1     1   Mm1
......................................................................aagcaccagguuauacUaguu.....................   1     1   Mm1
......................................................................aagcaccagguuauaccUguu.....................   1     1   Mm1
......................................................................aCgcaccagguuauaccaguuc....................   1     1   Mm1
......................................................................aagcaccagguuauaccaUuuc....................   1     1   Mm1
......................................................................aagcaccagguuauacGaguuc....................   11    1   Mm1
......................................................................aagcaccagguuauaccGguuc....................   27    1   Mm1
......................................................................aGgcaccagguuauaccaguuc....................   1     1   Mm1
......................................................................aagcGccagguuauaccaguuc....................   1     1   Mm1
......................................................................aagAaccagguuauaccaguuc....................   1     1   Mm1
......................................................................aagcaccaAguuauaccaguuc....................   1     1   Mm1
......................................................................aagcaccUgguuauaccaguuc....................   1     1   Mm1
......................................................................Nagcaccagguuauaccaguuc....................   5     1   Mm1
......................................................................aaAcaccagguuauaccaguuc....................   3     1   Mm1
......................................................................aagcaccagguCauaccaguuc....................   2     1   Mm1
......................................................................aaUcaccagguuauaccaguuc....................   2     1   Mm1
......................................................................aagcaccaggCuauaccaguuc....................   6     1   Mm1
......................................................................aagcaccagguuauUccaguuc....................   3     1   Mm1
```

```
                                Star                               Mature
uagaaguuguuuugucggauugcugcccagugcccuguuguauccugugugcuauugucaaaugaguucaaagcaccagguuauaccaguucauuuggcaugcaaauaaaua
......................................................................aagcaccagguuauaccUguuc....................   21     1   Mm1
......................................................................aagcaccagguuaAaccaguuc....................   4      1   Mm1
......................................................................Uagcaccagguuauaccaguuc....................   4      1   Mm1
......................................................................aagcaccaggAuauaccaguuc....................   13     1   Mm1
......................................................................aagcaccaggGuauaccaguuc....................   5      1   Mm1
......................................................................aagcaNcagguuauaccaguuc....................   1      1   Mm1
......................................................................aagcaccagguuUuaccaguuc....................   1      1   Mm1
......................................................................aagcaccagguuGuaccaguuc....................   1      1   Mm1
......................................................................aagcaccagAuuauaccaguuc....................   2      1   Mm1
......................................................................aagcaccagguuauaccaguCc....................   1      1   Mm1
......................................................................aagcacNagguuauaccaguuc....................   1      1   Mm1
......................................................................aagcaccagguuauaccCguuc....................   27     1   Mm1
......................................................................aagcaccagguuauacAaguuc....................   24     1   Mm1
......................................................................aagcaccagguuauaccaguuc....................   6477   0   Mm1
......................................................................aagcaccagguuauCccaguuc....................   1      1   Mm1
......................................................................aagcaccagguuauaccaguuU....................   3      1   Mm1
......................................................................aagcaccagguuauacUaguuc....................   14     1   Mm1
......................................................................aagcaccagguuauGccaguuc....................   1      1   Mm1
......................................................................aagcacUagguuauaccaguuc....................   2      1   Mm1
......................................................................aUgcaccagguuauaccaguuc....................   1      1   Mm1
......................................................................aagcaccaggAuauaccaguuca...................   1      1   Mm1
......................................................................aagcaccaggGuauaccaguuca...................   1      1   Mm1
......................................................................aagcaccagguuauacAaguuca...................   1      1   Mm1
......................................................................aagcaccagguuauaccCguuca...................   1      1   Mm1
......................................................................aagcaccagguuauacGaguuca...................   2      1   Mm1
......................................................................aagcaccagguuauaccUguuca...................   1      1   Mm1
......................................................................aagcaccagguuauaccaguucC...................   36     1   Mm1
......................................................................aagcaccagguuauaccGguuca...................   4      1   Mm1
......................................................................aagcaccagguuauacUaguuca...................   1      1   Mm1
......................................................................aagcaccagguuauaccaguuca...................   434    0   Mm1
......................................................................aagAaccagguuauaccaguuca...................   1      1   Mm1
......................................................................aagcaccagguuauaccaguucU...................   249    1   Mm1
......................................................................aagcaccagguuauaccaguucau..................   32     0   Mm1
......................................................................aagcaccagguuauaccaguucaA..................   26     1   Mm1
......................................................................aagcaccagguuauaccaguucaG..................   6      1   Mm1
......................................................................aagcaccagguuauaccaguucauu.................   2      0   Mm1
.......................................................................agcaccagguuauaccaguuc....................   1      0   Mm1
.......................................................................agcaccagguuauaccaguucU...................   3      1   Mm1

.................................ccuguuguauccugugugcu...........................................................   3      0   Fm2
.................................ccuguuguauAcugugugcuauu........................................................   1      1   Fm2
.................................ccuguuguauUcugugugcuauu........................................................   1      1   Fm2
.................................ccuguuguauccugugugcuauu........................................................   22     0   Fm2
.................................ccuguuguauccugugugcuauug.......................................................   34     0   Fm2
.................................ccuguuguauccuguguAcuauug.......................................................   1      1   Fm2
.....................................................................Caagcaccagguuauaccaguuc....................   2      1   Fm2
.....................................................................aaagcaccagguuauaccaguuc....................   8      0   Fm2
......................................................................aagcaccagguuauaccagu......................   4      0   Fm2
......................................................................aagcaccagguuauaccaUu......................   2      1   Fm2
......................................................................aagcaccagguuauaccaguu.....................   78     0   Fm2
......................................................................aagcaccagguuauaccCguu.....................   1      1   Fm2
......................................................................aagcaccagguuaAaccaguu.....................   1      1   Fm2
......................................................................aagcaccagguuauaUcaguuc....................   1      1   Fm2
......................................................................Nagcaccagguuauaccaguuc....................   6      1   Fm2
......................................................................aagcaccagguuauaccGguuc....................   34     1   Fm2
......................................................................aagAaccagguuauaccaguuc....................   1      1   Fm2
......................................................................Gagcaccagguuauaccaguuc....................   2      1   Fm2
......................................................................aagcacUagguuauaccaguuc....................   2      1   Fm2
......................................................................aagcaccagguuaAaccaguuc....................   1      1   Fm2
......................................................................aagcaccagguuauaccaguGc....................   1      1   Fm2
......................................................................aagcaccaCguuauaccaguuc....................   1      1   Fm2
......................................................................aagcaUcagguuauaccaguuc....................   1      1   Fm2
......................................................................aagcaccaggCuauaccaguuc....................   16     1   Fm2
......................................................................aagcaccagguuaCaccaguuc....................   2      1   Fm2
......................................................................aagcaccagguuGuaccaguuc....................   1      1   Fm2
......................................................................aagcaccagUuuauaccaguuc....................   1      1   Fm2
......................................................................aagcaccagguuauUccaguuc....................   1      1   Fm2
......................................................................aagcaccagguuauaccagAuc....................   2      1   Fm2
......................................................................aagcaccagguuauaccCguuc....................   30     1   Fm2
......................................................................aagcaNcagguuauaccaguuc....................   1      1   Fm2
```

Star	Mature			

uagaaguuguuuugucggauugcugcccagugcccuguuguauccugugugcuauugucaaaugaguucaaagcaccagguuauaccaguucauuuggcaugcaaauaaaua			
....aagcGccagguuauaccaguuc....................	1	1	Fm2
....aagcaccagguuUuaccaguuc....................	1	1	Fm2
....aagcaccagguuauGccaguuc....................	1	1	Fm2
....aagcCccagguuauaccaguuc....................	1	1	Fm2
....aagcaccagguuauacUaguuc....................	28	1	Fm2
....aagcaccagguuauacAaguuc....................	31	1	Fm2
....aagcacNagguuauaccaguuc....................	1	1	Fm2
....aagcaccagguuauaccaguuA....................	11	1	Fm2
....aagcaccaggAuauaccaguuc....................	19	1	Fm2
....aagcaccagguuauacGaguuc....................	9	1	Fm2
....aagcaccagguuauaccaguuG....................	1	1	Fm2
....aagcaccaggGuauaccaguuc....................	6	1	Fm2
....aagcaccagguuauaccaUuuc....................	1	1	Fm2
....aagcaccagguuauaccaguuc....................	8938	0	Fm2
....aagcaccagguuauaccUguuc....................	22	1	Fm2
....aagcacAagguuauaccaguuc....................	1	1	Fm2
....aagcaccCgguuauaccaguuc....................	1	1	Fm2
....aagcaccGgguuauaccaguuc....................	6	1	Fm2
....aagcaccagguGauaccaguuc....................	1	1	Fm2
....aagcaccagguuauaccagCuc....................	2	1	Fm2
....aGgcaccagguuauaccaguuc....................	2	1	Fm2
....aUgcaccagguuauaccaguuc....................	1	1	Fm2
....Uagcaccagguuauaccaguuc....................	5	1	Fm2
....aagcaccagguuauaccCguuca...................	5	1	Fm2
....aagcaccagguuauaccaAuuca...................	1	1	Fm2
....aagcaccagguuauaccUguuca...................	4	1	Fm2
....aagcaccagguuauaccaguuAa...................	2	1	Fm2
....aagcaccagguuauaccGguuca...................	1	1	Fm2
....aagcaccagguuGuaccaguuca...................	1	1	Fm2
....aagcaccagguuauacUaguuca...................	5	1	Fm2
....aagAaccagguuauaccaguuca...................	1	1	Fm2
....Nagcaccagguuauaccaguuca...................	1	1	Fm2
....aagcaccagguuauaccaguAca...................	1	1	Fm2
....aagcaccaAguuauaccaguuca...................	1	1	Fm2
....aagcaccagguuauaccaguucU...................	491	1	Fm2
....aagcaccagguuauaccaguuca...................	940	0	Fm2
....aagcaccaggAuauaccaguuca...................	1	1	Fm2
....aagcaccagguuauacAaguuca...................	4	1	Fm2
....aagcaccagguuauaccagCuca...................	2	1	Fm2
....aagcaccagguuauacGaguuca...................	1	1	Fm2
....aaAcaccagguuauaccaguuca...................	1	1	Fm2
....aagcaccagguuauaccaguucC...................	35	1	Fm2
....aCgcaccagguuauaccaguuca...................	1	1	Fm2
....aagcaccagguuauaccCguucau..................	1	1	Fm2
....aagcaccagguuauacAaguucau..................	2	1	Fm2
....aagcaccagguuauaccaguucUu..................	11	1	Fm2
....aagcaccagAuuauaccaguucau..................	1	1	Fm2
....aagcaccagguuauaccGguucau..................	1	1	Fm2
....aagcaccagguuauaccaguucau..................	58	0	Fm2
....aagcaccagguuauaccaguucaA..................	5	1	Fm2
....agcaccagguuauaccaguuc....................	3	0	Fm2
....agcaccagguuauaccaguucU...................	5	1	Fm2
....caccagguuauaccaguuc....................	3	0	Fm2
..........uuugucggauugcugcccagugc...	5	0	Fm1
.................................ccuguuguauccugugugcu...	1	0	Fm1
.................................ccuguuguauccuguguAcu...	1	1	Fm1
.................................ccuguuguauccugugugcuau...	2	0	Fm1
.................................ccuguuguauccugugugcuauu..	14	0	Fm1
.................................ccuguuguauccugugugcuauug...	17	0	Fm1
.................................ccuguCguauccugugugcuauug...	1	1	Fm1
.................................ccuguuguauccugugugcuCuug...	1	1	Fm1
....ucaaaugaguucaaagcaccagguuauaccagu......................	1	0	Fm1
....ucaaaugaguucaaagcaccagguuauaccaguuc....................	2	0	Fm1
....Caagcaccagguuauaccaguuc....................	6	1	Fm1
....aaagcaccagguuauaccaguuc....................	6	0	Fm1
....aagcaccagguuauacca........................	13	0	Fm1
....aagcaccagguuauaccag.......................	2	0	Fm1
....aagcaccagguuauaccagu......................	13	0	Fm1
....aagcaNcagguuauaccagu......................	1	1	Fm1

Star Mature

```
uagaaguuguuuugucggauugcugcccagugcccuguuguauccugugugcuauugucaaaugaguucaaagcaccagguuauaccaguucauuuggcaugcaaauaaaua
......................................................................aagcaccagguuauacAaguu.....................    2      1   Fm1
......................................................................aagcaccagguuauaccaguu.....................    92     0   Fm1
......................................................................aagcaccagguuauaccUguu.....................    1      1   Fm1
......................................................................aagcaccagguuauaccGguu.....................    1      1   Fm1
......................................................................aagcaccagguuauacAaguuc....................    62     1   Fm1
......................................................................aagcaccaCguuauaccaguuc....................    1      1   Fm1
......................................................................aagcaccUgguuauaccaguuc....................    1      1   Fm1
......................................................................aagcaccaggAuauaccaguuc....................    31     1   Fm1
......................................................................aagcaNcagguuauaccaguuc....................    4      1   Fm1
......................................................................Gagcaccagguuauaccaguuc....................    5      1   Fm1
......................................................................aagcaccagguuauaccaguuU....................    12     1   Fm1
......................................................................aagUaccagguuauaccaguuc....................    3      1   Fm1
......................................................................aagcaccagguuauUccaguuc....................    1      1   Fm1
......................................................................aagcaccaggGuauaccaguuc....................    19     1   Fm1
......................................................................aaUcaccagguuauaccaguuc....................    3      1   Fm1
......................................................................aagcaccaUguuauaccaguuc....................    2      1   Fm1
......................................................................aagcaccagguuauaccUguuc....................    37     1   Fm1
......................................................................aagcacAagguuauaccaguuc....................    3      1   Fm1
......................................................................aaCcaccagguuauaccaguuc....................    6      1   Fm1
......................................................................aagcaccGgguuauaccaguuc....................    17     1   Fm1
......................................................................aagcaccagUuuauaccaguuc....................    1      1   Fm1
......................................................................aagcaccaggCuauaccaguuc....................    16     1   Fm1
......................................................................aagcaccagguuUuaccaguuc....................    1      1   Fm1
......................................................................aagcCccagguuauaccaguuc....................    2      1   Fm1
......................................................................aUgcaccagguuauaccaguuc....................    3      1   Fm1
......................................................................aagcaccagguuauacUaguuc....................    32     1   Fm1
......................................................................Uagcaccagguuauaccaguuc....................    9      1   Fm1
......................................................................aagcaccagguCauaccaguuc....................    2      1   Fm1
......................................................................aagcaccagguuauacGaguuc....................    25     1   Fm1
......................................................................aagcaccagguGauaccaguuc....................    2      1   Fm1
......................................................................aagcaccagguuauaccaguGc....................    1      1   Fm1
......................................................................aagcaccagguuauaUcaguuc....................    4      1   Fm1
......................................................................aagGaccagguuauaccaguuc....................    1      1   Fm1
......................................................................aagAaccagguuauaccaguuc....................    8      1   Fm1
......................................................................aagcaccagguuauaccGguuc....................    54     1   Fm1
......................................................................aagcUccagguuauaccaguuc....................    2      1   Fm1
......................................................................aagcaccagguuauaccCguuc....................    33     1   Fm1
......................................................................aagcaccagguuauaccaUuuc....................    3      1   Fm1
......................................................................aagcaUcagguuauaccaguuc....................    4      1   Fm1
......................................................................aagcaccagguuauGccaguuc....................    6      1   Fm1
......................................................................aagcaccagguuauaccaguuA....................    10     1   Fm1
......................................................................aaAcaccagguuauaccaguuc....................    3      1   Fm1
......................................................................aCgcaccagguuauaccaguuc....................    1      1   Fm1
......................................................................Nagcaccagguuauaccaguuc....................    5      1   Fm1
......................................................................aagcGccagguuauaccaguuc....................    3      1   Fm1
......................................................................aagcaccagguuGuaccaguuc....................    1      1   Fm1
......................................................................aagcaccagguuauaccaguuc....................    13386  0   Fm1
......................................................................aagcacUagguuauaccaguuc....................    5      1   Fm1
......................................................................aagcaccagguuauaccaguAc....................    1      1   Fm1
......................................................................aagcaccagguuaGaccaguuc....................    1      1   Fm1
......................................................................aGgcaccagguuauaccaguuc....................    2      1   Fm1
......................................................................aagcaccaggAuauaccaguuca...................    4      1   Fm1
......................................................................aagcaccagguuauaccUguuca...................    7      1   Fm1
......................................................................aagcaccagguuauaccCguuca...................    5      1   Fm1
......................................................................aagcUccagguuauaccaguuca...................    1      1   Fm1
......................................................................aagcaccGgguuauaccaguuca...................    1      1   Fm1
......................................................................aagcaccagguuaCaccaguuca...................    1      1   Fm1
......................................................................aagcaccagguuauaccagAuca...................    1      1   Fm1
......................................................................aagcaccagguuauaccGguuca...................    1      1   Fm1
......................................................................aagcaccagguuauaccaguuca...................    1292   0   Fm1
......................................................................aagcaccagguuauGccaguuca...................    1      1   Fm1
......................................................................aagcaccagguuauacAaguuca...................    5      1   Fm1
......................................................................aagcaNcagguuauaccaguuca...................    1      1   Fm1
......................................................................aagcaccagguuauaccaguucU...................    745    1   Fm1
......................................................................aagcaccagguuauaccaguucC...................    85     1   Fm1
......................................................................aagcaccagAuuauaccaguuca...................    1      1   Fm1
......................................................................aagcaccagguuauacUaguuca...................    7      1   Fm1
......................................................................aagcaccagguuauacGaguuca...................    5      1   Fm1
......................................................................aagcaccagguuauaccaguuUau..................    1      1   Fm1
......................................................................aagcaccagguuauaccUguucau..................    1      1   Fm1
```

Star Mature

```
uagaaguuguuuugucggauugcugcccagugcccuguuguauccugugugcuauugucaaaugaguucaaagcaccagguuauaccaguucauuuggcaugcaaauaaaua
......................................................................aagcaccagguuauaccaguucaG..................  5  1  Fm1
......................................................................aagcaccagguuauaccaguucaA..................  38  1  Fm1
......................................................................aagcaccagguuauacUaguucau..................  1  1  Fm1
......................................................................aagcaccagguuauaccaguucau..................  72  0  Fm1
......................................................................aagcaccagguuauaccaguucaC..................  8  1  Fm1
......................................................................aagcaccagguuauaccaguucCu..................  5  1  Fm1
......................................................................aagcaccagguuauaccaguucUu..................  49  1  Fm1
......................................................................aagcaccagguuauaccaguucauu.................  5  0  Fm1
......................................................................aagcaccagguuauaccaguucUuu.................  8  1  Fm1
......................................................................aagcaccagguuauaccaguucaCu.................  1  1  Fm1
......................................................................aagcaccagguuauaccaguucauA.................  2  1  Fm1
......................................................................aagcaccagguuauaccaguucaAu.................  1  1  Fm1
.......................................................................agcaccagguuauaccaguuc....................  2  0  Fm1

..............ucggauugcugccUagug................................................................................  1  1  Mm3
.................gauuUcugcccagugccc.............................................................................  2  1  Mm3
.................gauuUcugcccagugcccug...........................................................................  1  1  Mm3
................................cccuguuguauccugugu..............................................................  1  0  Mm3
................................cccuguuguauccugugugcu...........................................................  21  0  Mm3
................................cccuguuguauccugugugcuU..........................................................  2  1  Mm3
................................cccuguuguauccugugCgcua..........................................................  1  1  Mm3
................................cccuguuguauccugugugcua..........................................................  2  0  Mm3
................................cccuguuguauUcugugugcuau.........................................................  1  1  Mm3
................................cccuguuguauccugugugcuau.........................................................  16  0  Mm3
................................cccuguuguauccugugugcuauu........................................................  25  0  Mm3
................................cccuguuguauccugugugcuauug.......................................................  9  0  Mm3
.................................ccuguuguauccugugugcu...........................................................  1  0  Mm3
.................................ccuguuguauccugugugcua..........................................................  1  0  Mm3
.................................ccuguuguauccugugugcuau.........................................................  5  0  Mm3
.................................ccuguuguauccugugugcuauA........................................................  1  1  Mm3
.................................ccuguuguauccugugugcuauu........................................................  19  0  Mm3
.................................ccuguuguauccugugugcuauug.......................................................  45  0  Mm3
.................................ccuguuguauccugugGgcuauug.......................................................  1  1  Mm3
.................................ccuguuguauccuguguCcuauug.......................................................  1  1  Mm3
.................................ccuguuguauccugugugcuauugG......................................................  1  1  Mm3
.................................ccuguuguauccugugugcuauugA......................................................  1  1  Mm3
.................................ccuguuguauccugugugcuauugucaaaugaguuca..........................................  1  0  Mm3
.........................................................ucaaaugaguucaaagcaccagguu..............................  1  0  Mm3
.....................................................................aaagcaccagguuauaccaguu.....................  2  0  Mm3
.....................................................................aaagcaccagguuauaccaguuc....................  1  0  Mm3
.....................................................................Caagcaccagguuauaccaguuc....................  2  1  Mm3
.....................................................................Caagcaccagguuauaccaguuca...................  1  1  Mm3
......................................................................aagcaccagguuauacca........................  5  0  Mm3
......................................................................aagcaccagguuauaccagu......................  10  0  Mm3
......................................................................aagcaccagguuauaccaguu.....................  104  0  Mm3
......................................................................aagcaccagguuauacAaguu.....................  1  1  Mm3
......................................................................aagcaccagguuauaccaAuu.....................  1  1  Mm3
......................................................................Uagcaccagguuauaccaguuc....................  4  1  Mm3
......................................................................aagcaccaggGuauaccaguuc....................  6  1  Mm3
......................................................................Cagcaccagguuauaccaguuc....................  1  1  Mm3
......................................................................aagcaccagguuauaccaguuU....................  9  1  Mm3
......................................................................aagAaccagguuauaccaguuc....................  1  1  Mm3
......................................................................aaAcaccagguuauaccaguuc....................  1  1  Mm3
......................................................................aagcaccagguuauaccUguuc....................  9  1  Mm3
......................................................................aagcaccagguuauaccCguuc....................  24  1  Mm3
......................................................................aagcaUcagguuauaccaguuc....................  1  1  Mm3
......................................................................aagcaccagguuauacGaguuc....................  13  1  Mm3
......................................................................aagcaccagguuauacUaguuc....................  30  1  Mm3
......................................................................aagcaccagguuauacAaguuc....................  21  1  Mm3
......................................................................aagcaccagguuauaccagCuc....................  1  1  Mm3
......................................................................aagcaccaAguuauaccaguuc....................  1  1  Mm3
......................................................................aaUcaccagguuauaccaguuc....................  1  1  Mm3
......................................................................aagcaccGgguuauaccaguuc....................  2  1  Mm3
......................................................................aagcaccagguuauaUcaguuc....................  1  1  Mm3
......................................................................Gagcaccagguuauaccaguuc....................  2  1  Mm3
......................................................................aagcaccagUuuauaccaguuc....................  2  1  Mm3
......................................................................aagcaccaggCuauaccaguuc....................  7  1  Mm3
......................................................................aagcaccagguuauaccaguuc....................  5641  0  Mm3
......................................................................aagcaccagguuauUccaguuc....................  2  1  Mm3
......................................................................aagcaNcagguuauaccaguuc....................  2  1  Mm3
```

Star **Mature**

```
uagaaguguuuugucggauugcugcccagugcccuguuguauccugugugcuauugucaaaugaguucaaagcaccagguuauaccaguucauuuggcaugcaaauaaaua
....................................................................aagUaccagguuauaccaguuc....................  1     1  Mm3
....................................................................aagcaccagguuauaccGguuc....................  21    1  Mm3
....................................................................aagcaccaUguuauaccaguuc....................  1     1  Mm3
....................................................................aagcaccagguuauaccaguuA....................  3     1  Mm3
....................................................................aagcaccagguuaAaccaguuc....................  1     1  Mm3
....................................................................aagcaccagguuauaccaguCc....................  1     1  Mm3
....................................................................aagcaccagguuaCaccaguuc....................  2     1  Mm3
....................................................................aagcaccaggAuauaccaguuc....................  14    1  Mm3
....................................................................aagcacUagguuauaccaguuc....................  3     1  Mm3
....................................................................aagcaccagguuauaccaUuuc....................  1     1  Mm3
....................................................................Nagcaccagguuauaccaguuc....................  1     1  Mm3
....................................................................aagcaccagguuauGccaguuc....................  2     1  Mm3
....................................................................aagcaccUgguuauaccaguuc....................  2     1  Mm3
....................................................................aUgcaccagguuauaccaguuc....................  3     1  Mm3
....................................................................aagcaccagguuauaccaguuca...................  1147  0  Mm3
....................................................................aagcaccGgguuauaccaguuca...................  1     1  Mm3
....................................................................aagcaccaggCuauaccaguuca...................  1     1  Mm3
....................................................................aagcaccaggGuauaccaguuca...................  2     1  Mm3
....................................................................aagcaccagguuauacGaguuca...................  1     1  Mm3
....................................................................aagcaccagguuauacAaguuca...................  3     1  Mm3
....................................................................aagcaccagguuauaccCguuca...................  6     1  Mm3
....................................................................aagcacNagguuauaccaguuca...................  1     1  Mm3
....................................................................aagcaccagguuauaccaguuAa...................  1     1  Mm3
....................................................................aagcaccagguuauaccGguuca...................  6     1  Mm3
....................................................................aagcaccagguuauaccaguucU...................  290   1  Mm3
....................................................................Uagcaccagguuauaccaguuca...................  2     1  Mm3
....................................................................aagcaccagguuauacUaguuca...................  2     1  Mm3
....................................................................aagcaccagguuauaccUguuca...................  4     1  Mm3
....................................................................aagcaccaggAuauaccaguuca...................  2     1  Mm3
....................................................................aagcaccagguuauaccaguucC...................  4     1  Mm3
....................................................................aaAcaccagguuauaccaguuca...................  2     1  Mm3
....................................................................aagcaUcagguuauaccaguuca...................  1     1  Mm3
....................................................................aagcaccagguuauaccaguucau..................  42    0  Mm3
....................................................................aagcaccagguuauaccaguucUu..................  7     1  Mm3
....................................................................aagcaccagguuauaccaguucCu..................  1     1  Mm3
....................................................................aagcaccagguuauaccaguucaA..................  27    1  Mm3
....................................................................aagcaccagguuauaccaguucaG..................  3     1  Mm3
....................................................................aagcaccagguuauaccaguucaAu.................  3     1  Mm3
....................................................................aagcaccagguuauaccaguucauu.................  7     0  Mm3
.....................................................................agcaccagguuauaccaguuc....................  7     0  Mm3
.....................................................................agcaccagguuauaccaguucaC..................  1     1  Mm3
......................................................................gcaccagguuauaccaguuc....................  1     0  Mm3
.........................................................................ccagguuauaccaguuca...................  1     0  Mm3

...............................ccuguuguauccugugugcu...........................................................  9     0  Fm3
...............................ccuguuguauccugugugcuau.........................................................  1     0  Fm3
...............................ccuguuguauccugugugcuauu........................................................  9     0  Fm3
...............................ccuguuguauccugugugcuauug.......................................................  55    0  Fm3
...............................ccuguuguauccugugugcuauuU.......................................................  1     1  Fm3
...................................................................Caagcaccagguuauaccaguuc....................  1     1  Fm3
....................................................................aagcaccagguuauacca........................  19    0  Fm3
....................................................................aagcaccagguuauaccaU.......................  5     1  Fm3
....................................................................aagcaccagguuauaccCg.......................  2     1  Fm3
....................................................................aagcaccagguuauaccag.......................  195   0  Fm3
....................................................................aagcaccGgguuauaccag.......................  1     1  Fm3
....................................................................aagcaccaUguuauaccag.......................  1     1  Fm3
....................................................................aagcaccagguuauacAag.......................  2     1  Fm3
....................................................................aGgcaccagguuauaccag.......................  1     1  Fm3
....................................................................aagcaccagguuauaccagu......................  27    0  Fm3
....................................................................aagcaccagguuauaccagC......................  1     1  Fm3
....................................................................Uagcaccagguuauaccagu......................  1     1  Fm3
....................................................................aagcaccagguuauaccUgu......................  1     1  Fm3
....................................................................aagcaccagguuauaUcagu......................  3     1  Fm3
....................................................................aagcaccagguuauGccaguu.....................  1     1  Fm3
....................................................................aagAaccagguuauaccaguu.....................  1     1  Fm3
....................................................................aagcaccagguuauaccUguu.....................  2     1  Fm3
....................................................................aagcaccagguuauaccGguu.....................  2     1  Fm3
....................................................................aagcaccagguuauaccaguu.....................  334   0  Fm3
....................................................................aagcaAcagguuauaccaguu.....................  1     1  Fm3
....................................................................aagcaccagguuauacGaguu.....................  1     1  Fm3
```

```
                                Star                                Mature
uagaaguuguuuugucggauugcugcccagugcccuguuguauccugugugcuauugucaaaugaguucaaagcaccagguuauaccaguucauuuggcaugcaaauaaaua
......................................................................aagcaccagguuauacAaguu.....................   2      1   Fm3
......................................................................aagcaccagguGauaccaguu.....................   2      1   Fm3
......................................................................aagcaccaggGuauaccaguu.....................   1      1   Fm3
......................................................................aagcaccagguuauacUaguu.....................   2      1   Fm3
......................................................................Gagcaccagguuauaccaguu.....................   1      1   Fm3
......................................................................aagcaccaggAuauaccaguu.....................   2      1   Fm3
......................................................................aagcaccagguuauacGaguuc....................   2      1   Fm3
......................................................................aagcacAagguuauaccaguuc....................   2      1   Fm3
......................................................................aagcaccagguuauacAaguuc....................   12     1   Fm3
......................................................................aaUcaccagguuauaccaguuc....................   1      1   Fm3
......................................................................aagcaccagguuauaccaguGc....................   1      1   Fm3
......................................................................aagcaccagguuauacUaguuc....................   10     1   Fm3
......................................................................aagcaccagguuauaccCguuc....................   4      1   Fm3
......................................................................Gagcaccagguuauaccaguuc....................   1      1   Fm3
......................................................................aagcaccagguuauaccaguuc....................   2489   0   Fm3
......................................................................aagAaccagguuauaccaguuc....................   2      1   Fm3
......................................................................aaAcaccagguuauaccaguuc....................   1      1   Fm3
......................................................................aagcaccagguuauaccUguuc....................   12     1   Fm3
......................................................................aagcaccagguuauaccaguuG....................   1      1   Fm3
......................................................................aagcaccGgguuauaccaguuc....................   1      1   Fm3
......................................................................aagUaccagguuauaccaguuc....................   1      1   Fm3
......................................................................aagcaccaggGuauaccaguuc....................   3      1   Fm3
......................................................................aagcaccagguuauaccagAuc....................   2      1   Fm3
......................................................................aagcaccagguuGuaccaguuc....................   1      1   Fm3
......................................................................aagcaccagguuaCaccaguuc....................   1      1   Fm3
......................................................................aCgcaccagguuauaccaguuc....................   1      1   Fm3
......................................................................aagcaccagguuaAaccaguuc....................   1      1   Fm3
......................................................................aagcaccaggCuauaccaguuc....................   2      1   Fm3
......................................................................aagcaccagguuauaccaguAc....................   1      1   Fm3
......................................................................aagcaccagguCauaccaguuc....................   2      1   Fm3
......................................................................aagcaccagguuauaccGguuc....................   10     1   Fm3
......................................................................aagGaccagguuauaccaguuc....................   2      1   Fm3
......................................................................aagcaccaggAuauaccaguuc....................   9      1   Fm3
......................................................................aaCcaccagguuauaccaguuc....................   1      1   Fm3
......................................................................aagcaccagguuauaccaguuU....................   5      1   Fm3
......................................................................aagcaccagguuauaUcaguuc....................   1      1   Fm3
......................................................................aagcaccagguuauaccaguucC...................   3      1   Fm3
......................................................................aagcaccaggCuauaccaguuca...................   2      1   Fm3
......................................................................aagcaccaggAuauaccaguuca...................   2      1   Fm3
......................................................................aagcaccagguuauacGaguuca...................   1      1   Fm3
......................................................................aagcaccagguuauaccCguuca...................   1      1   Fm3
......................................................................aagcaccagguuauaccaguucU...................   74     1   Fm3
......................................................................aagcaccagguuauacAaguuca...................   3      1   Fm3
......................................................................aagcacGagguuauaccaguuca...................   1      1   Fm3
......................................................................aagcaccagguuauaccUguuca...................   1      1   Fm3
......................................................................aagcaccagguuauaccaguuca...................   400    0   Fm3
......................................................................aagcaccaggGuauaccaguuca...................   1      1   Fm3
......................................................................aagcaccagguuauaccGguuca...................   1      1   Fm3
......................................................................aagcaccagguuauaccaguucUu..................   1      1   Fm3
......................................................................aagcaccagguuauacGaguucau..................   1      1   Fm3
......................................................................aagcaccagguuauaccaguucaG..................   4      1   Fm3
......................................................................aagcaccagguuauaccaguucau..................   23     0   Fm3
......................................................................aagcaccagguuauaccaguucaA..................   15     1   Fm3
......................................................................aagcaccagguuauaccaguucauu.................   2      0   Fm3
.......................................................................agcaccagguuauaccaguuc....................   1      0   Fm3
```
